# Seasonality of Cat-Scratch Disease, France, 1999–2009

**DOI:** 10.3201/eid1704.100825

**Published:** 2011-04

**Authors:** Diane Sanguinetti-Morelli, Emmanouil Angelakis, Hervé Richet, Bernard Davoust, Jean Marc Rolain, Didier Raoult

**Affiliations:** Author affiliations: Université de la Méditerranée, Marseille, France (D. Sanguinetti-Morelli, E. Angelakis, H. Richet, J.M. Rolain, D. Raoult);; Service de Santé des Armées–Secteur Vétérinaire, Marseille (B. Davoust)

**Keywords:** Seasonality, cat-scratch disease, cats, zoonoses, Bartonella henselae, bacteria, France, dispatch

## Abstract

Cat-scratch disease is seasonal in the United States and Japan; but no data are available from Europe. To assess the seasonality of the disease in France, we analyzed lymph node biopsy specimens collected during 1999–2009. Most (87.5%) cases occurred during September–April and peaked in December.

*Bartonella henselae* is the causative agent of cat-scratch disease (CSD), the most common cause of lymphadenopathy in adults and children (*1*). Cats are the main reservoir of *B. henselae*, which is transmitted among cats by the *Ctenocephalides felis* flea (*2*). *Bartonella* organisms remain viable in flea feces, and transmission to humans results in inoculation of *B. henselae*–contaminated flea feces into the skin through a scratch (*3*). However, transmission of *B. henselae* from cats to humans through scratches is rare (*4*). In classic CSD, gradual regional lymph node enlargement is accompanied by a papule that develops in the scratch line after 3–10 days and persists from a few days to 2–3 weeks (*4*).

The link between seasons and CSD incidence has been described in the United States (*5**,**6*) and in Japan (*7*). However, because no data are available on seasonal variations of CSD in Europe, or in France, we studied lymph node biopsy specimens obtained January 1999–December 2009 from patients throughout France with suspected CSD.

## The Study

Tissue specimens were sent to the National Reference Center (Marseilles, France) either frozen or in transport media. CSD was definitively diagnosed when a specimen was positive for 2 genes of *Bartonella* spp (*1*). Total genomic DNA was extracted from samples by using a QIAamp tissue kit (QIAGEN, Hilden, Germany). Before 2005, PCR amplification and sequencing of the internal transcribed spacer (ITS) region and *pap31* gene were used for detecting *B. henselae* and thus confirming CSD (*1*). Beginning in 2005, real-time PCR to amplify the ITS region and *pap31* gene was used (*8*). For all assays, 2 sets of negative controls were used. DNA from *B*. *elizabethae* and *B*. *henselae* Houston-I was used as the positive control for the ITS region and the *pap31* gene, respectively (*1*). To exclude false-positive results, we performed a second independent extraction when false-positive or unexpected results were obtained. Results were confirmed by using PCR amplification and sequencing aimed at 16S rRNA gene (*8*).

Epi Info version 6.0 software (Centers for Disease Control and Prevention, Atlanta, GA, USA) was used for significance variations in the number of positive specimens between 2 consecutive months, nonconsecutive months, and seasons (p<0.05). The Mantel-Haenszel test or the Fisher exact test was used to test for significance.

**Figure 1:**
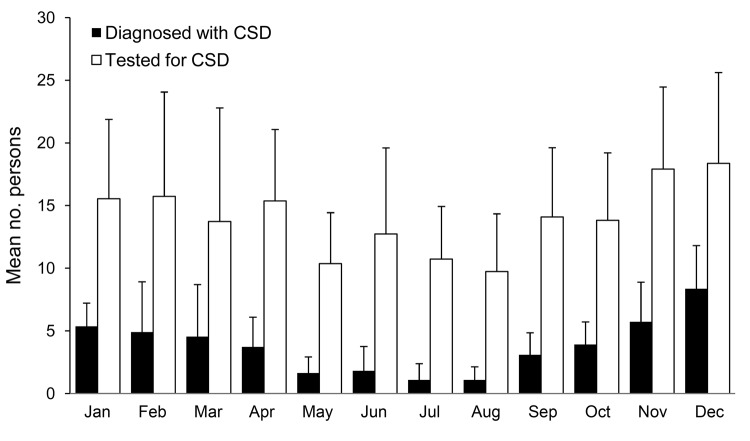
Mean numbers of patients tested for cat-scratch disease and for whom the disease was diagnosed, France, 1999–2009. Error bars indicate 95% confidence intervals.

We tested 1,849 lymph node biopsy specimens and identified *B*. *henselae* in 493. Positive and negative controls yielded the expected results in all tests. Positive CSD cases were plotted for each month (Figure 1) to identify seasonal distributions of CSD from 1999 through 2009. Monthly mean numbers of CSD were lowest from May through August, followed by significant increases during August–September (p = 0.002) and during November–December (p = 0.01). During December–January, the mean number of CSD cases decreased significantly (p = 0.005), then plateaued from January through March (Table). Cases decreased slightly in April, then decreased significantly during April–May (p = 0.002).

**Table:** Monthly number of patients with cat-scratch disease, France, 1999–2009

Month	Mean no. patients	Median no. patients	SD
January	5.4	6	1.86
February	4.9	6	4
March	4.6	2	4.2
April	3.7	4	2.4
May	1.6	1	1.3
June	1.8	1	1.9
July	1.1	1	1.3
August	1.1	1	1
September	3.1	3	1.8
October	3.9	4	1.8
November	5.7	5	3.2
December	8.4	7	3.4

The odds of a CSD diagnosis based on lymph node biopsy was 9.2× higher in December, the month with the highest number of cases (92/493), than in July, the month with the lowest number of cases (12/493). The number of CSD cases was significantly higher in autumn (October–December) than in summer (July–September) (p<0.0001). Fewer cases were identified in winter (January–March) than autumn (p = 0.02), and cases decreased significantly from winter to spring (April–June) (p = 0.0001). The number of cases did not differ significantly from spring to summer (p = 0.06).

## Conclusions

Our findings that the number CSD cases in France varies by season are similar to findings in Japan and the United States. In Japan, 64% of CSD occurred during September–December and peaked in November (*7*). In the United States, most CSD have occurred during the last 6 months of the year, with a peak in September (*9*). Moreover, the analysis of 3 US national databases indicated that most CSD cases have occurred during September–January, with peaks in November and December (*5*). On the other hand, 60% of admissions for CSD in children in the United States have occurred during July–October (*6*). The fact that the United States is a large country with diverse climates, whereas continental France has a more homogeneous climate, may explain the differences in seasonality.

**Figure 2:**
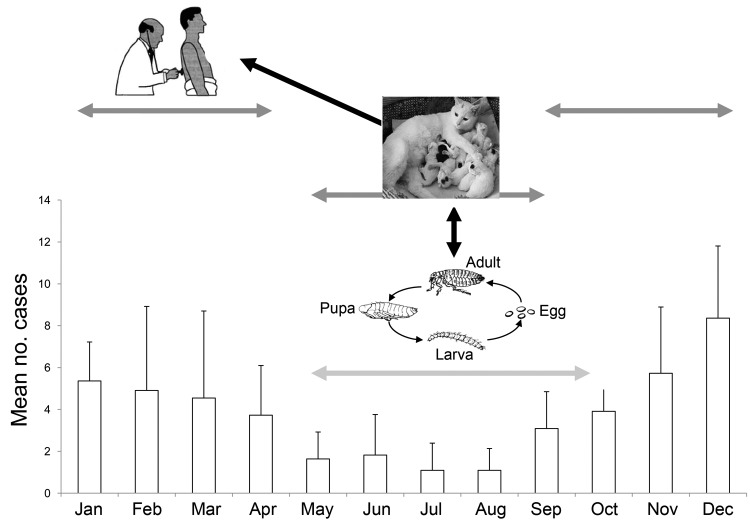
Explanation of cat scratch disease seasonality by seasonality of the birth of cats and the activity of their fleas, France, 1999–2009. Error bars indicate 95% confidence intervals.

The presence of *Ct. felis* fleas is essential for maintaining *B. henselae* infection within the cat population (*2*). Flea infestation is more frequent in bacteremic than in nonbacteremic cats, particularly in pet cats (*10*). After adult cat fleas parasitize a host cat, they feed on its blood and transmit *B. henselae*. Fleas go through 4 life cycle stages: egg, larva, pupa, and imago (adult) (Figure 2). Temperature and relative humidity are the 2 most essential factors for the successful reproduction, development, and survival of fleas (*11*). Cats reported to have been infested with fleas during the preceding 6 months were more likely than cats without fleas to be seropositive (*12*), and the seroprevalence of *B. henselae* is higher in the pet cat population in warm, humid climates than in cold, dry climates because *Ct. felis* fleas are more common in warmer climates (*10*). As a result, cats have more fleas during the summer and autumn months than in the other 2 seasons (*13*).

In Nancy, France, 53% of 94 stray cats were infected with either *B. henselae* or *B. clarridgeiae* (*14*). In Paris, Chomel et al. reported a *B. henselae* seroprevalence of 36% in 64 pet cats, of which 11% were *B. henselae* infected (*12*). Gurfield et al. determined that 16.5% of cats tested were *Bartonella* infected, and 41% were seropositive for *B. henselae* or *B. clarridgeiae* (*15*). Risk for *Bartonella* infection or seropositivity was higher in cats from multicat households and in cats adopted from the pound or from the street (*15*).

Feline sexual activity also may influence the seasonality of CSD. In the Northern Hemisphere, cat reproduction increases during spring and summer, and kittens stay with their mothers until they are 12–16 weeks of age. In addition, humans are more likely to acquire kittens during the autumn months. *B. henselae* infection appears to be more common in young cats (*10*), and infection decreases with the length of cat ownership (*15*). In addition, cats encounter more fleas during summer and autumn, and transmission of *B. henselae* from cat to cat is facilitated during this period (*13*).

In conclusion, CSD is a seasonal disease in France with increased incidence in autumn, with peaks in December, and a decrease in spring. This pattern may be explained by seasonality in cat reproductive behavior, their fleas’ activities, and the fact that during summer cats spend most time outside the house, whereas during autumn they stay indoors.

## References

[1] Rolain JM, Lepidi H, Zanaret M, Triglia JM, Michel G, Thomas PA, Lymph node biopsy specimens and diagnosis of cat-scratch disease. Emerg Infect Dis. 2006;12:1338–44. 17073081 10.3201/eid1209.060122 PMC3294744

[2] Chomel BB, Kasten RW, Floyd-Hawkins K, Chi B, Yamamoto K, Roberts-Wilson J, Experimental transmission of *Bartonella henselae* by the cat flea. J Clin Microbiol. 1996;34:1952–6. 8818889 10.1128/jcm.34.8.1952-1956.1996 PMC229161

[3] Finkelstein JL, Brown TP, O’Reilly KL, Wedincamp J Jr, Foil LD. Studies on the growth of *Bartonella henselae* in the cat flea (Siphonaptera: Pulicidae). J Med Entomol. 2002;39:915–9. 10.1603/0022-2585-39.6.915 12495192

[4] Ridder GJ, Boedeker CC, Technau-Ihling K, Grunow R, Sander A. Role of cat-scratch disease in lymphadenopathy in the head and neck. Clin Infect Dis. 2002;35:643–9. 10.1086/342058 12203159

[5] Jackson LA, Perkins BA, Wenger JD. Cat scratch disease in the United States: an analysis of three national databases. Am J Public Health. 1993;83:1707–11. 10.2105/AJPH.83.12.1707 8259799 PMC1694941

[6] Reynolds MG, Holman RC, Curns AT, O’Reilly M, McQuiston JH, Steiner CA. Epidemiology of cat-scratch disease hospitalizations among children in the United States. Pediatr Infect Dis J. 2005;24:700–4. 10.1097/01.inf.0000172185.01939.fc 16094224

[7] Tsukahara M. Cat scratch disease in Japan. J Infect Chemother. 2002;8:321–5. 10.1007/s10156-002-0202-x 12525891

[8] Angelakis E, Roux V, Raoult D, Rolain JM. Real-time PCR strategy and detection of bacterial agents of lymphadenitis. Eur J Clin Microbiol Infect Dis. 2009;28:1363–8. 10.1007/s10096-009-0793-6 19685089

[9] Carithers HA. Cat-scratch disease: an overview based on a study of 1,200 patients. Am J Dis Child. 1985;139:1124–33. 4061408 10.1001/archpedi.1985.02140130062031

[10] Chomel BB, Abbott RC, Kasten RW, Floyd-Hawkins KA, Kass PH, Glaser CA, *Bartonella henselae* prevalence in domestic cats in California: risk factors and association between bacteremia and antibody titers. J Clin Microbiol. 1995;33:2445–50. 7494043 10.1128/jcm.33.9.2445-2450.1995 PMC228433

[11] Dryden MW, Rust MK. The cat flea: biology, ecology and control. Vet Parasitol. 1994;52:1–19. 10.1016/0304-4017(94)90031-0 8030176

[12] Chomel BB, Gurfield AN, Boulouis HJ, Kasten RW, Piemont Y. Réservoir félin de l’agent de la maladie des griffes du chat, *Bartonella henselae*, en région Parisienne: résultats préliminaires. Rec Med Vet Ec Alfort. 1995;171:841–5.

[13] Farkas R, Gyurkovszky M, Solymosi N, Beugnet F. Prevalence of flea infestation in dogs and cats in Hungary combined with a survey of owner awareness. Med Vet Entomol. 2009;23:187–94. 10.1111/j.1365-2915.2009.00798.x 19712149

[14] Heller R, Artois M, Xemar V, De Briel D, Gehin H, Jaulhac B, Prevalence of *Bartonella henselae* and *Bartonella clarridgeiae* in stray cats. J Clin Microbiol. 1997;35:1327–31. 9163438 10.1128/jcm.35.6.1327-1331.1997 PMC229743

[15] Gurfield AN, Boulouis HJ, Chomel BB, Kasten RW, Heller R, Bouillin C, Epidemiology of *Bartonella* infection in domestic cats in France. Vet Microbiol. 2001;80:185–98. 10.1016/S0378-1135(01)00304-2 11295338

